# Polystyrene Topography Sticker Array for Cell-Based Assays

**DOI:** 10.21926/rpm.2002013

**Published:** 2020-05-14

**Authors:** Heizel Rosado-Galindo, Maribella Domenech

**Affiliations:** 1. Mayagüez Campus-Bioengineering Program, University of Puerto Rico, Mayagüez, Puerto Rico; 2. Mayagüez Campus-Department of Chemical Engineering, University of Puerto Rico, Mayagüez, Puerto Rico

**Keywords:** Cell culture, xurography, topography, razor printing, cell behavior, polystyrene

## Abstract

Cells can respond to different topographical cues in their natural microenvironment. Hence, scientists have employed microfabrication techniques and materials to generate culture substrates containing topographies for cell-based assays. However, one of the limitations of custom topographical platforms is the lack of adoption by the broad research community. These techniques and materials have high costs, require high technical expertise, and can leach components that may introduce artifacts. In this study, we developed an array of culture surfaces on polystyrene using razor printing and sanding methods to examine the impact of microscale topographies on cell behavior. The proposed technology consists of culture substrates of defined roughness, depth, and curvature on polystyrene films bound to the bottom of a culture well using double-sided medical-grade tape. Human monocytes and adult mesenchymal stem cells (hMSCs) were used as test beds to demonstrate the applicability of the array for cell-based assays. An increase in cell elongation and Arg-1 expression was detected in macrophages cultured in grooves and on rough substrates as compared to flat surfaces. Also, substrates with enhanced roughness stimulated the proliferation of hMSCs. This effect correlated with the secretion of proteins involved in cell proliferation and the downregulation of those associated with cell differentiation. Our results showed that the polystyrene topography sticker array supports cellular changes guided by microscale surface roughness and geometries. Consequently, microscale surface topographies on polished and razor-printed polystyrene films could leverage the endogenous mechanisms of cells to stimulate cellular changes at the functional level for cell-based assays.

## 1. Introduction

Cells can sense the physical features of their microenvironment, such as topography, rigidity, and density of the extracellular matrix (ECM) [1-3]. The mechanisms underlying the interactions in cell topography are not fully understood, but they can greatly influence cell proliferation [4], cytoskeletal organization [5], differentiation [6, 7], and receptor signaling [8]. For example, studies found that micro- and nano-patterned grooves influence human macrophages elongation, and stimulate an anti-inflammatory and pro-healing phenotype [9, 10]. Other *in vitro* studies also demonstrated that mechanical strain [11], nano-topographies [12], surface roughness [13, 14], and substrate stiffness [13] play important roles in the differentiation of stem cells for tissue engineering and in regenerative medicine. Hence, the design of surface topographies could guide changes in cell behavior.

Natural polymers, such as hydrogels, collagen fibers, and Matrigel [15], are frequently molded into substrates to generate surface patterns with desired physical properties for cellular studies. Compared to other biomaterials, these polymers have increased biocompatibility and similar physical properties to natural tissue [16], which enable successful interaction of the cell with the surface interface. Yet, they are more expensive than their synthetic counterparts, are prone to animal-derived contaminants [17-19], and degrade over time. These cause the loss of the desired physical properties [17], in turn, limiting the endpoint analysis to a short time window. Also, dense substrates, such as electrospun collagen fibers, have reduced optical transparency and background fluorescence, which limit cell imaging applications and other optical-based assays. On the contrary, synthetic polymers, including polydimethylsiloxane (PDMS), poly (ε-caprolactone) (PCL), and olefin copolymer (OC), are effective in generating topographical cues in cell culture substrates [20-23]. For example, PDMS is one of the most commonly used materials for microfabricated topographies. It is biocompatible, has low toxicity, can be molded to a nanometer resolution, and is optically transparent [24]. However, it releases oligomers into the cell culture solution and sequesters small hydrophobic molecules to the polymer bulk, which have an impact on biological results [25]. Similarly, PCL is used for biomedical applications since it is biocompatible and non-toxic [26]. However, it degrades over time and has low bioactivity, which makes it inadequate to examine the desired properties [27].

Among synthetic polymers, polystyrene (PS) is the current gold standard surface for adherent cell cultures. It is preferred over other materials due to its biocompatibility and physical properties. PS is stable over time, non-toxic to the cells, provides optical clarity, easy to manufacture, and has low production costs [28]. Common topographies generated on PS are nano- and micro-grooves [29, 30] and TopoChip-derived features [31, 32], generated using nano- and microfabrication methods [29, 31-34]. Hot embossing [35, 36] is the most frequently used technique to fabricate polystyrene topographies, performed in a two-step process. First, a master mold is generated using lithography-based fabrication-nanoimprint lithography, UV assisted lithography, or photolithography [37]. Second, the devices are replicated from the master mold using hot embossing. There are other alternative methods for polystyrene molding, such as laser cutting, 3D printing [38], micromilling [38, 39], and thermal scribing [40]. These techniques enable the generation of topographical cues on rigid surfaces in a controlled and reproducible fashion suitable for large-scale operations [41]. However, most of them require a high initial investment for instrumentation (> $10,000) or lack commercial availability. Lithography-based techniques need clean facilities, are low-throughput for prototyping time (> 48 hrs), and require a high level of technical expertise; whereas, methods that generate heat (e.g., laser cutting) can produce toxic byproducts by the combustion process. These limitations have contributed to the lack of broad adoption and the designing of custom culture arrays among research laboratories [42]. Furthermore, methods that employ laser cutters or chemical etching generate chemical byproducts that can be toxic to cells or induce an undesirable response that can lead to artifacts. Thus, other strategies for user-friendly, biocompatible, and fast fabrication of PS-based culture arrays are desirable for high-throughput cell-based studies at the basic research level.

One way to address this fabrication barrier is to employ conventional, user-friendly, and commercially available instrumentation for surface modification of rigid polymers at the microscale level. For example, we [42], as well as others [44-46], successfully employed razor printing or xurography [43] to generate culture platforms made of PS at the microscale resolution. Further, they were also used for geometric patterning of electrospun substrates for cell applications [45]. The razor printer employs a razor blade to cut or “print” polymer sheets into the desired shapes. Chandrasekaran employed a similar system to generate microfluidic channels instead of topographies using a thermal tip instead of the razor blade [40]. Sanding is another method to modify rigid surfaces physically without the need for heat. It uses rudimentary tools, such as sandpaper or sandblasting, to generate a range of surface roughnesses [47-49].

Although razor printing and sanding methods are well-known in the microfluidic community, none of these have been examined for the generation of topographical culture arrays on rigid polymers. In this study, we employed razor printing and sanding methods to generate substrates of defined roughness, depth, and curvature on polystyrene adhesive films. We validated the substrates generated for cell culture applications using two cell lines known to be responsive to surface topographies. Our results demonstrated that cells perceive microscale changes in surface roughness and geometries. Also, these changes have a measurable effect on cell growth, morphology, and phenotype/secretome.

## 2. Materials and Methods

### 2.1 PS Substrate Design, Fabrication, and Assembly

We followed the methodology for the generation of razor-printed sticker-like devices, as previously described [42]. Briefly, razor printing was performed with a cutting plotter (CE6000-40 Plus, Graphtec America, USA) equipped with a 0.9 mm diameter and 60° angle Graphtec blade (CB09UA). We used a medical-grade tape (ARCare 90106), consisting of a clear polyester substrate coated on both sides with MA-69 acrylic hybrid medical-grade adhesive. The tape (without its protective backings) has a total thickness of 143 ± 14.3 μm. We used a biaxially oriented, 0.19 mm thick polystyrene (PS) film (ST311190/3, Goodfellow). The PS film and the tape can be obtained directly as sheets or rolls for razor printing. We drew the designs using the Graphtec Studio software. For the substrate assembly, a PS sheet was first applied to one side of the tape. This PS–Tape laminate was then taped to a folder and fed through the cutting plotter to cut the desired pattern. Then, the PS sticker-like substrates were taped to the bottom of a 96-well culture plate and plasma-treated using a corona plasma treater (BD20-ACV) to increase the hydrophilicity of the surface and promote cell attachment. Before cell culture, the plates were sterilized using UV light and washed twice with 1× phosphate buffer saline (PBS), each with a 15 min exposure to UV light. The cell culture medium was added overnight to ensure sterility.

### 2.2 Characterization

#### 2.2.1 Grooves

We generated grooved polystyrene substrates by setting a specific cutting force for the razor blade in the control panel of the razor printer software. This cutting force was varied from 1 to 13 in increments of two. A 3D laser scanning microscope (VK-X-1000, Keyence) was used to scan the samples, and groove depth was measured using the Keyence Multi File analyzer software.

#### 2.2.2 Roughness

Polystyrene films with surface roughness were generated using an in-house polishing device. Sandpapers were adapted to a homemade compression instrument with two stainless steel plaques fixed to an adjusting screw. A sandpaper sheet was mounted on to the top plaque of the instrument. Then, a PS film sheet of approximately 2″ × 8″ was placed between the top and bottom plaques. Next, the top plaque was adjusted to a fixed pressure using the adjusting screw. Finally, the PS sheet was manually pulled out of the device, generating the roughness pattern in the polystyrene sheet.

### 2.3 Cell Culture

For cell culture experiments, we used mesenchymal stem cells (hMSC): bone marrow-derived (female, 26 years) and adipose-derived (female, 18-30 years) and THP-1 cells, purchased from RoosterBio and American Type Culture Collection (ATCC), respectively. The hMSC cells were maintained in DMEM high glucose medium with L-glutamine (D5796, Sigma-Aldrich), supplemented with 10% heat-inactivated fetal bovine serum (FBS) (F6765, Sigma-Aldrich), 1% penicillin–streptomycin (P4333, Sigma-Aldrich), and 1% NEAA (M7145, Sigma Aldrich). The THP-1 cells were maintained in RPMI-1640 medium (R7388, Sigma-Aldrich), supplemented with 10% heat-inactivated fetal bovine serum (FBS) (F6765, Sigma-Aldrich), 1% penicillin–streptomycin (P4333, Sigma-Aldrich), and 0.05 mM 2-mercaptoethanol. All cells were cultured at 37 °C in 5% CO_2_ and were mycoplasma-free. The hMSC passages were performed at 75-80% confluence using 0.25% trypsin (59418C, Sigma-Aldrich). The hMSCs were used below seven passages, and the THP-1 cells were used below 40 passages. Viable cells were counted using a hemocytometer and the trypan blue exclusion method (T8154, Sigma–Aldrich). The differentiation of THP-1 monocytes to macrophages occurred after treatment with 18 nM of phorbol 12-myristate 13-acetate (1585, Sigma-Aldrich) for 12 hrs. Macrophage M2 polarization was performed using 20 ng/mL of IL-13 and 20 ng/mL of IL-4 for 72 hrs.

### 2.4 Fluorescence Staining and Microscopy

For fluorescence microscopy, cells were fixed in 4% paraformaldehyde (50-980-487, Fisher Scientific) in PBS for 15 min. Then, they were permeabilized using 0.2% Triton X-100 (Sigma–Aldrich) in PBS for 20 min at room temperature. The actin cytoskeleton was stained with ActinRed (2 drops/mL of PBS; R37112, Invitrogen) for 30 min protected from light at room temperature. Similarly, the DNA was stained with 1:1000 diluted Hoechst 33342 (16.2 mM; Invitrogen) for 10 min protected from light at room temperature. Fluorescent images were taken using a fluorescence microscope (BZ-X800, Keyence).

### 2.5 Cell Morphology

Image analysis of hMSCs, seeded at a density of 6,250 cells/cm^2^, was performed using ImageJ software. Cells were manually traced, and the major and minor axes were calculated using the “fit ellipse” measurement in ImageJ. Cell elongation factor was calculated as the ratio between the major axis and the minor axis of the cell. We analyzed 60 cells per condition for each independent experiment. Similarly, image analysis of THP-1 cells, seeded at 100,000 cells/cm^2^, was carried out as described above.

### 2.6 Immunofluorescence Staining

The THP-1 cells were seeded on the PS substrates at a density of 100,000 cells/cm^2^ for arginase-1 (Arg-1) staining. Samples were fixed with 4% paraformaldehyde (Electron Microscopy Sciences) for 15 min. Then, the cells were permeabilized with 0.5% Triton X-100 in PBS for 10 min, washed twice, and blocked with 3% BSA with 0.1% Tween-20 for 1 h at room temperature. Cells were then incubated with the primary antibody, mouse anti-arginase-I (Santa Cruz Biotechnology Inc.), in a 3% BSA solution with 0.1% Tween-20 for 1.5 h, washed thoroughly with PBS 0.1% Tween-20, and incubated with the secondary antibody, Alexa Fluor-647 goat anti-mouse (Abcam), for 1 h. All cells were counterstained with Hoechst 33342, washed thoroughly, and then imaged using the BZ-X800 fluorescence microscope under a 20 × objective. Fluorescence intensity was analyzed using ImageJ. Cells were manually selected. Integrated density is the sum of the intensities of all the pixels in the cells. It was used as the level of Arg-1 expression. At least 60 cells per condition were examined for each independent experiment.

### 2.7 Statistics

Multifactorial analysis using ANOVA was performed to identify significant changes between the conditions. Tukey’s multiple comparisons were also implemented to compare changes with baseline values or treatment controls. Results were deemed statistically significant for combined *P* < 0.05 for at least three independent experiments with n = 3-6 per condition.

## 3. Results

### 3.1 Characterization of Polystyrene Topographical Array

**Figure 1:**
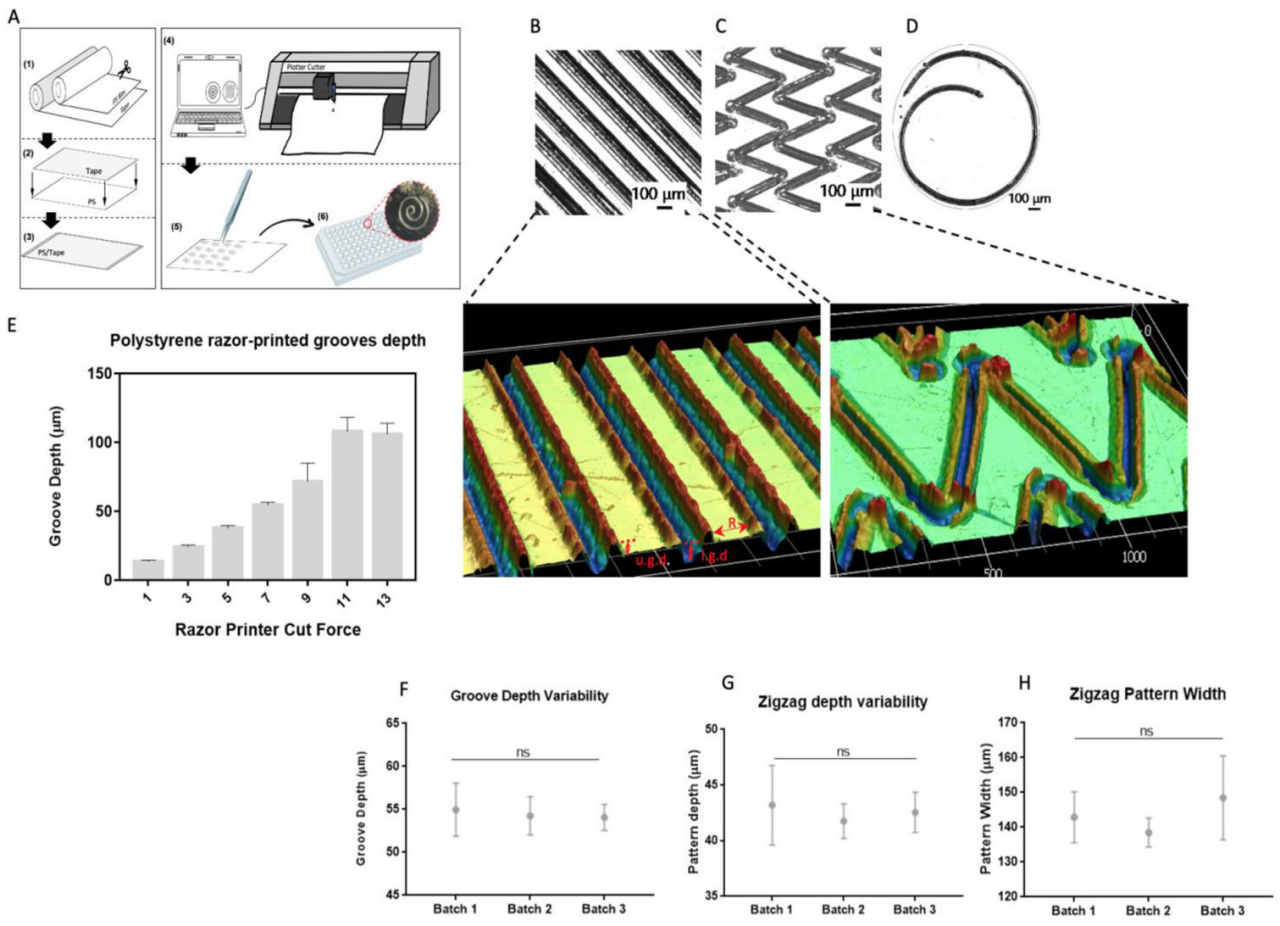
Razor-printing micropatterning and characterization. (A) Generation of razor-printed micropatterns in polystyrene films. (1) PS sheet and double-sided tape were cut to the desired size, then (2) the PS sheet was attached to the double-sided tape, and (3) used in the razor-printer to (4) cut the micropatterns. (5) The razor-printed micropatterns were peeled-off and (6) taped to the bottom of a 96-well culture plate and sterilized afterward. Brightfield and 3D images of (B) 55 μm depth grooves, (C) zigzags, and (D) spiral micropatterns. © Quantification of the depth of razor-printed grooves. Batch-to-batch variability for (F) depth of cut force 7 grooves (Average characterized depth = 55μm), and zigzag micropattern (G) depth and (H) width. Average values were calculated based on 12 samples; error bars represent the standard deviation. Scale bar of 3D images: μm.

The following three topographical features were evaluated for the polystyrene substrate array: geometry, depth, and surface roughness. The selection of substrates was based on topographies previously described in the literature [10, 50, 51]. The array included razor-printed micropatterns consisting of zigzags, spirals, and grooves generated on polystyrene films to assess the effect of corners, curvatures, and depth, respectively, in cell function (Figure 1A). It is important to note that the characterization discussed in this section corresponds to a 96-well culture plate with diameter (D) = 0.6 cm and area (A) = 0.32 cm^2^. Spiral micropatterns had an average outer diameter of 2.8 mm ± 0.4 mm (Figure 1D). Zigzag patterns were approximately 4.5 mm in height and 4.5 mm in width (Figure 1C). Both spiral and zigzag micropatterns had an average depth of 40 μm ± 14 μm and a width of 143.3 μm ± 47 μm. The grooves had a ridge width © of 75 μm ±10 μm. The average groove depth denoted as lower groove depth (l.g.d.) in Figure 1B, correlated with the cutting force used (Figure 1E). The razor blade cut on the polystyrene surface created a slope between the edges of the grooves and the ridge that resembled another groove. Thus, we termed these regions as secondary grooves. The secondary grooves had a depth between 11 μm and 37 μm, depending on the employed cutting force (denoted as upper groove depth, u.g.d. in Figure 1B). Additionally, we examined the variability and reproducibility of the razor-printed substrates (Figure 1F, G, H). Average groove depth, zigzag depth, and zigzag width were measured and compared among three independent batches with no significant difference between them. Groove depths (l.g.d.) of 55 μm (cut force = 7), 70 μm (cut force = 9), and 106 μm (cut force = 13) were selected for examination in cells.

**Figure 2:**
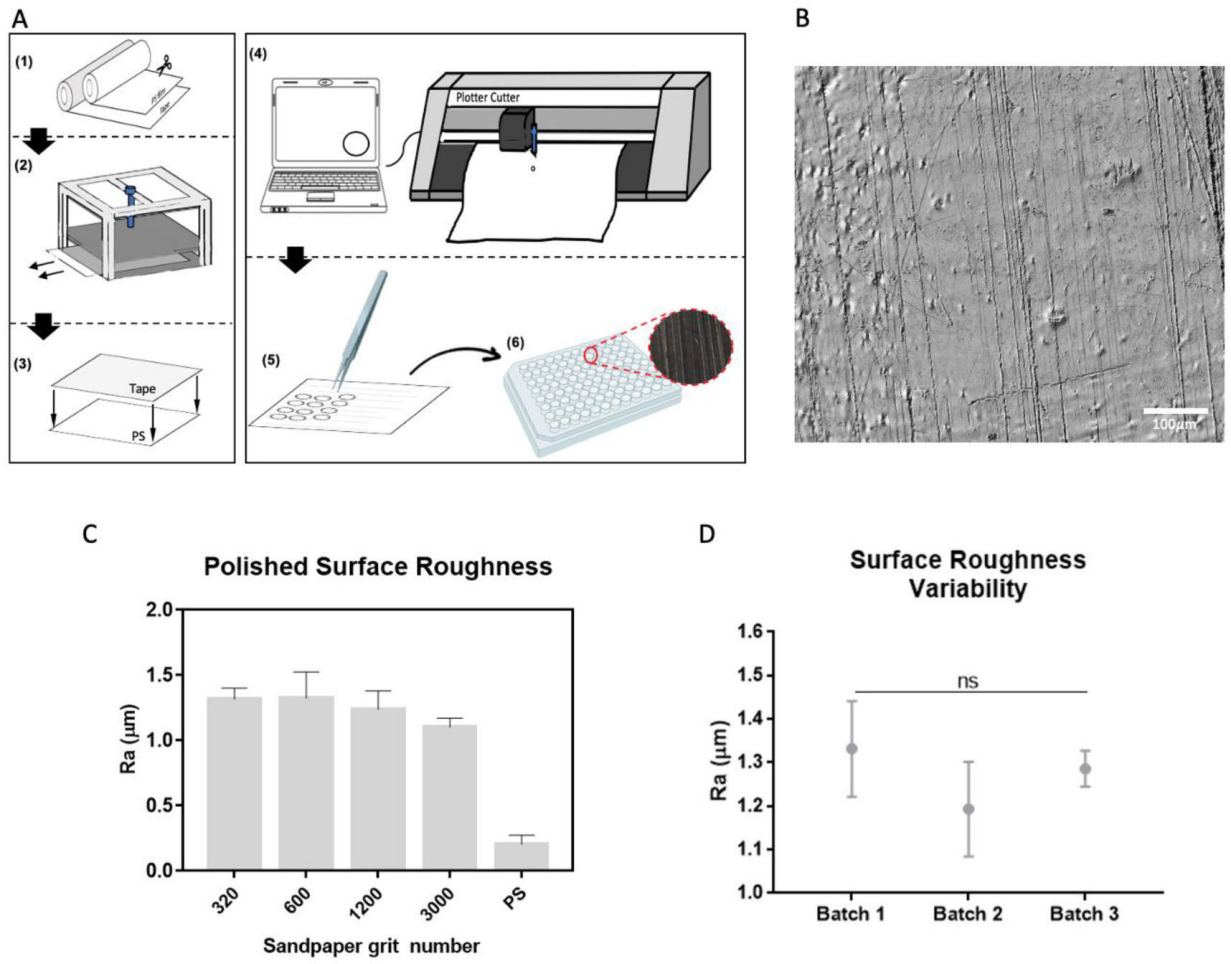
Surface roughness fabrication and characterization. (A) Generation of polished PS films. (1) The PS sheet and double-sided tape were cut to the desired size, then (2) the PS sheet was polished using the in-house designed polisher. Next, (3) the polished PS sheet was attached to the double-sided tape, and (4) cut into 6 mm diameter circles (96-well dimensions). (5) The PS substrates were peeled-off and (6) taped to the bottom of a 96-well culture plate and sterilized afterward. (B) Brightfield image of a low roughness (3000 grit, Ra = 1.18μm) polished PS film. (C) Quantification of the average profile roughness (Ra) of polished PS films. (D) Batch-to-batch variability for surfaces with a high level of roughness (sandpaper grit 600, Ra = 1.5μm). Average values were calculated based on nine samples; error bars represent the standard deviation.

Polished surfaces with different levels of roughness were developed on PS films using sandpaper of coarse, medium, and fine grits, specifically, 320, 600, 1200, and 3000 grits. Figure 2A displays the set-up used for making the patterns; Figure 2B shows the pattern generated when the PS sheet was manually pulled out of the device.

Surface roughness (Ra) was measured using the VK-X-1000 3D laser microscope (Keyence). The sandpaper generated a microscale roughness inversely proportional to the grit number. Thus, the lower the sandpaper grit number, the higher the average roughness. Average roughness (Ra) values ranged from 0.2 μm to 1.3 μm (Figure 1C). PS film roughness was used as a control for low to non-roughness levels. Based on this information, we selected substrates with non-overlapping standard deviation values for the culture array. The following three surface roughness levels were selected and considered as high, medium, and low roughness substrates, respectively: 600 (Ra = 1.3 μm), 3000 (Ra = 1.18 μm), and raw PS (Ra = 0.2 μm).

### 3.2 PS Grooves and Roughness Modulate Macrophage M2 Phenotype

**Figure 3:**
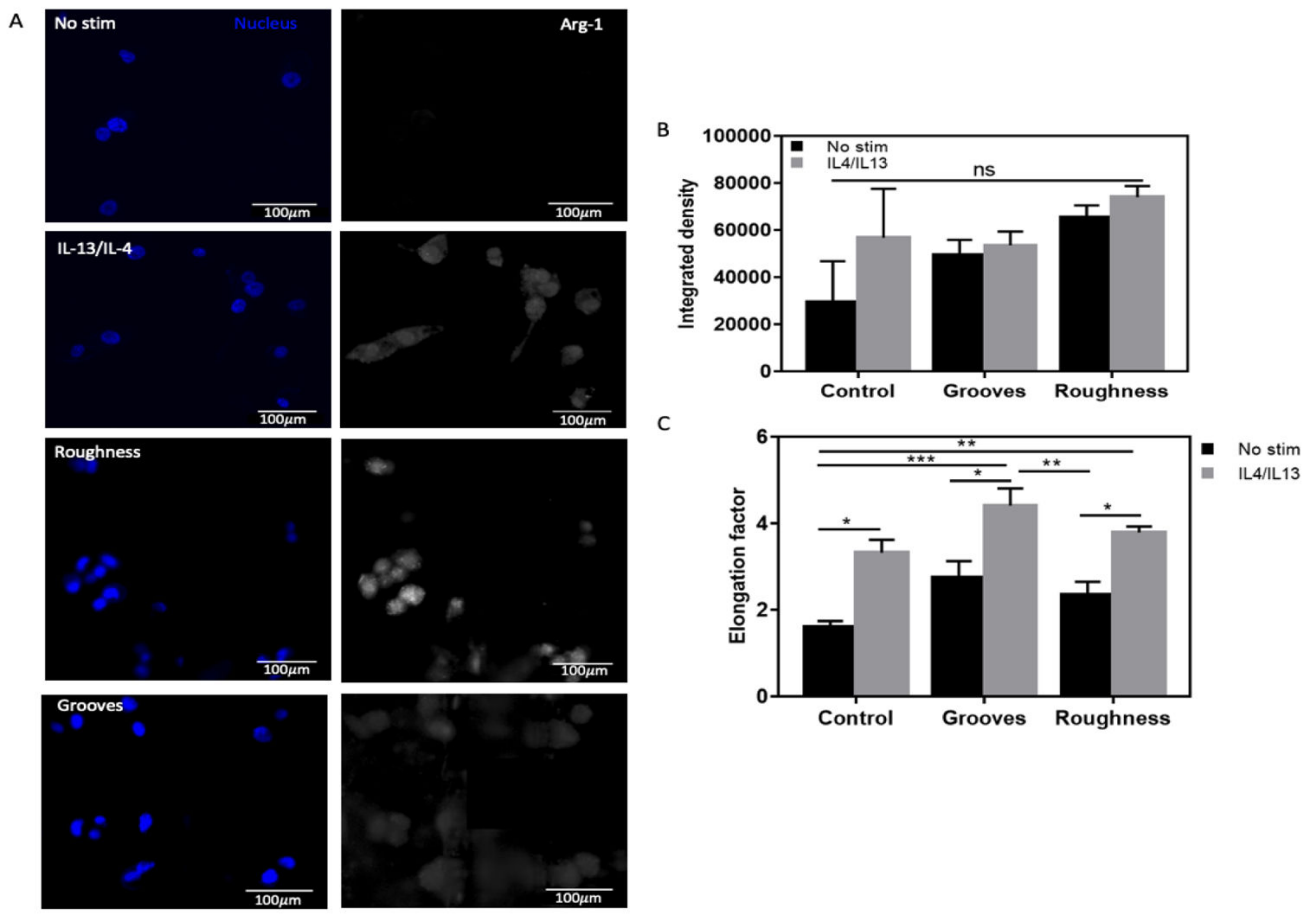
Effect of PS topographical array in macrophage M2 polarization. Representative Arg-1 fluorescent micrographs of macrophages differentiated from THP-1 cells after 72 hrs of culture on the PS 55μm depth grooves, high roughness substrates (600 grit, Ra = 1.3μm) and controls. (B) Quantification of Arg-1 expression by the integrated density, which represents the sum of all the pixel intensities in the selected cells. (C) Quantification of the macrophage elongation factor on the PS 55 μm depth grooves, high roughness substrates and controls. Error bars represent standard deviation (One-way ANOVA, n = 2).

One potential application of this array is to induce mechanical stimulus by generating changes in the cell cytoskeleton caused by confinement to a particular culture surface pattern. Hence, we examined a macrophage M2 polarization model [10, 50] in our array to validate its potential application. The THP–1 macrophages were cultured in substrates of high roughness (Ra = 1.3 μm) and 55 μm depth grooves (cut force = 7) with or without exogenous cytokine stimulus for M2 polarization. Cell elongation and arginase-1 (Arg-1) expression were measured, since they are a morphological hallmark [52] and a marker [53] of M2 polarized macrophages, respectively. Macrophages were more aligned along the grooves and, thus, more elongated compared to the other substrates. Arg-1 expression determined if these changes in morphology correlated with this molecular marker of the M2 phenotype. Results showed higher Arg-1 expression on substrates with roughness compared to substrates with grooves and the tissue culture plastic plate (control) (Figure 3). However, there was no significant difference between them. Interestingly, there was no observable correlation between cell elongation and Arg-1 expression. In fact, macrophages cultured in rough substrates were less elongated, yet, expressed higher levels of Arg-1. Nevertheless, the cells expressed Arg-1 in both substrates (grooves and roughness), consistent with the previous studies [10, 50]. Thus, our data suggest that topographical arrays can promote cytoskeletal changes perceived at the cellular level.

### 3.3 Effect of PS Topographical Array in Morphology, Proliferation, and Secretome of hMSCs

**Figure 4:**
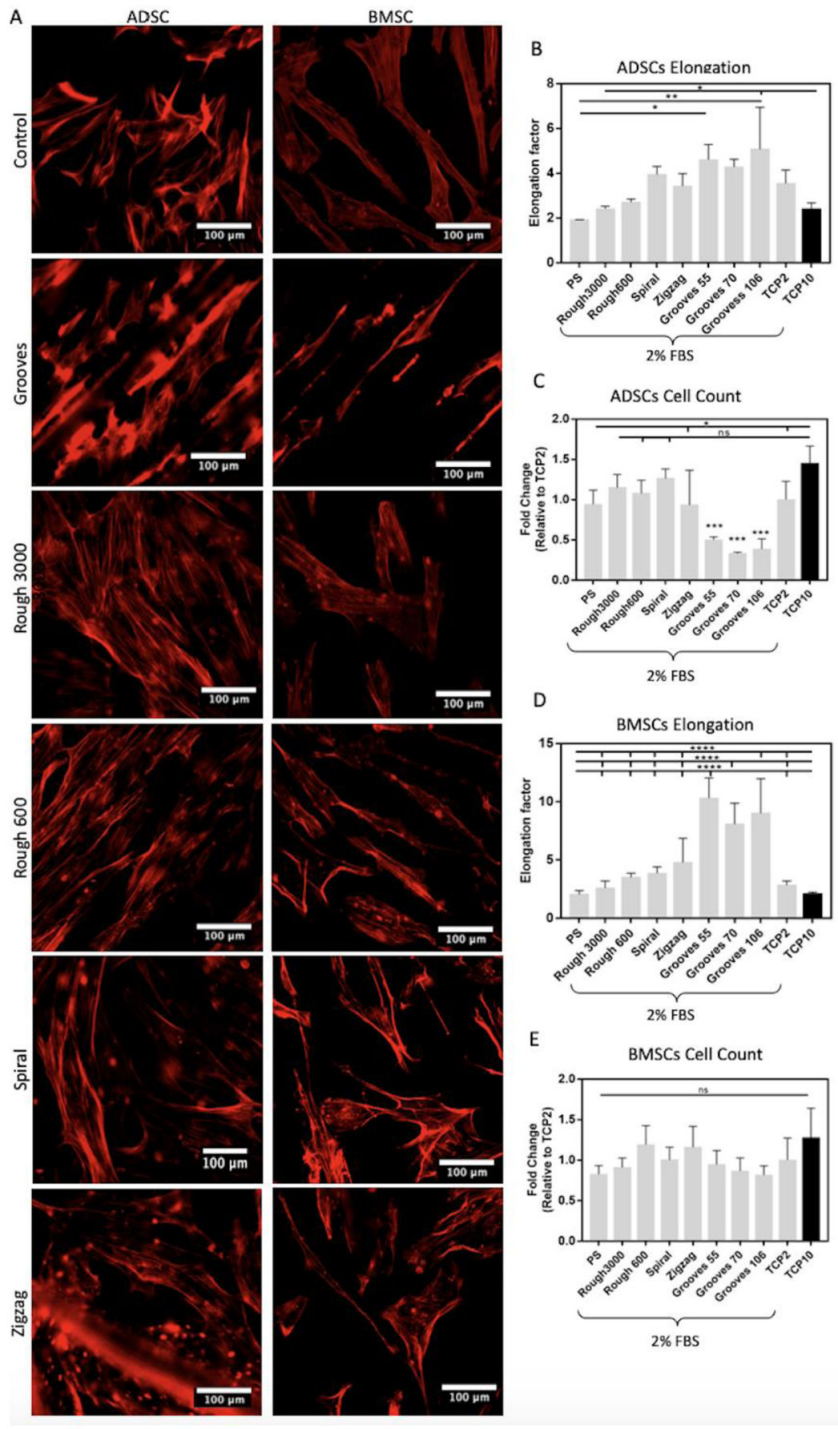
Effect of PS topographical array in hMSC morphology and proliferation. (A) Fluorescent microscopic images of hMSC grown in tissue culture plastic (control), grooves, low roughness level (Rough3000), high roughness level (Rough600), spiral and zigzag micropatterns stained with actin red for the identification of the cytoskeleton. (B) Quantification of ADSCs elongation factor in all the substrates. (C) Quantification of ADSCs total cell count. (D) Quantification of BMSCs elongation factor in all the substrates. (E) Quantification of BMSCs total cell count. Sixty cells were counted per substrate to calculate the average cell elongation. Error bars represent the standard deviation. (One-way ANOVA, n = 3).

Human adult mesenchymal stem cells (hMSCs) are also sensitive to biomechanical stimulation [53-56]. Surface stiffness is one of the well-known properties [57]; however, the impact of other surface properties, such as roughness and geometrical patterns, on cell function is less understood. The hMSCs derived from bone marrow and adipose tissues were cultured on the topographical array for five days, using reduced serum (2% FBS) to minimize its effect on cell growth. We fixed the cells and analyzed the images to quantify cell growth and morphology. Visual analysis of the fluorescence micrographs confirmed different cell morphologies induced by the topographies (Figure 4A). Elongation of the adipose tissue-derived stem cells (ADSCs) significantly increased when cultured in the grooves (Figure 4B). However, the proliferation of cells on these substrates dropped significantly. Nevertheless, the proliferation of ADSCs was enhanced on substrates with roughness, including spiral micropatterns, showing no significant difference between the substrates with roughness or curvature and the control. This suggests that the physical stimulus provided by these substrates is comparable to the control (10% FBS) (Figure 4C). Likewise, grooves also significantly increased the elongation of bone marrow-derived stem cells (BMSCs); yet, cell proliferation in these substrates was comparable to tissue culture plastic (TCP) plates (Figure 4D). We also noted that the substrates with roughness had higher elongation than TCP, suggesting that BMSCs are more mechanosensitive than ADSCs. Additionally, the proliferation of BMSCs was either maintained or enhanced in the PS substrates, although there was no significant difference as compared to TCP (Figure 4E). This, too, suggests that the physical stimulus provided by these substrates is comparable to the control (10% FBS).

We performed a preliminary assessment of 440 human inflammatory and growth factors, chemokines, receptors, and cytokines to confirm the impact of surface roughness at the secretome level. The ADSCs expressed several growth factors and cytokines when cultured on substrates with a high level of roughness but not on tissue culture plastic, such as IL-17F, EVGF-C, MCSF, FGF-19, EGF-R, midkine, MIF, GCP-2, Singlec-9, BMCA, INFab R2, IL-1 R6, and MIP-1b [57, 58]. Similarly, proteins related to the anti-proliferative activity (e.g., ferritin) were suppressed in substrates with high levels of roughness (Figure S1) [57, 58]. Along with the experimental results obtained from macrophages, these data further validate the use of topographical PS array generated by razor printing and physical polishing to guide cell changes at the cell cytoskeleton and secretome levels.

## 4. Discussion

One of the limitations of custom microscale devices for cell-based assays is the lack of adoption by the non-engineering research community. Fabrication methods used in this field require high costs of investment and depend on multiple steps that demand the supervision of specialized and highly trained personnel. These discourage broad adoption and potential for technology innovation across disciplines. We had previously validated a tape-based razor printing approach as a low cost and fast strategy for prototyping of microscale culture platforms [42]. Here, our study expands the potential of razor printing in combination with macro surface polishing to generate topographical arrays on PS films. Specifically, we employed razor printing and surface polishing as alternative methods to the classical hot embossing approach. We invested less than $2,000 for the instrumentation, including a razor printer, PS sheets, adhesive rolls, and polishing instrument parts. Compared to hot embossing and lithography methods, the advantages of our proposed method are: (1) no requirement for a mask or mold, (2) fast prototyping time (1 h), and (3) affordability [42]. Also, it requires low technical expertise to generate this topographical array on PS, equivalent to using a paper laser printer. Thus, non-engineering laboratories could easily adapt such methods. Furthermore, unlike natural polymers, PS retains its physical and chemical properties after prolonged shelf storage (> 1 year) and during culture due to its natural resistance to environmental conditions and cell remodeling. This enables pre-fabrication of the substrates and investigation of physical cell stimulation on cell behavior for extended periods (> 7 days).

We could use razor-printed and polished topographies on PS films for cell mechanosensing on well-based culture platforms. Our results showed that cells perceive the microscale changes in surface roughness and geometries on PS substrates. Also, these changes have a measurable effect on cell growth, morphology, and phenotype/secretome. Previous studies demonstrated that microgrooved patterns favor stem-like phenotype [59]. Thus, substrates that favor cell elongation could enhance or retain the pluripotent capacity of the cells. Also, we observed that roughness increased the proliferation of hMSCs. This effect correlated with the secretion of growth factors and proteins involved in proliferation and suppression of differentiation molecules. Thus, surface topographies could stimulate changes at the secretome level to favor a cell behavior, while minimizing the need for exogenous biochemical supplementation in both, in vitro cultures and potency assays. Moreover, as shown in our previous studies [42, 45], the proposed fabrication approach and the substrate materials used make these substrates easily adaptable into the microscale culture platforms. This would aide in incorporating topographical cues within culture microenvironments of enhanced biological complexities, while retaining the compatibility with standard optical methods, molecular assays, and high-throughput endpoint analyses.

It is important to note that the proposed approach for making patterns on PS films is limited to microscale dimensions due to the inherent capabilities of the instrumentation used. The observed batch-to-batch variability was not significant, and cellular behavior was reproducible across experiments. Despite these, the variability within each batch favors its application toward microscale topographical features and low volume fabrication. For a scaled-up process, the variability across batches may increase significantly as the razor blade and polishing tools are susceptible to wear-off in a short period. These could be circumvented by frequent replacement of razor blades and by using polishing tools resistant to wear-off (e.g., metal-based tools), thus minimizing such variability for scaled-up applications.

Although nanoscale topographies are not discussed in this study, these features are likely to be present and may have an impact on cell behavior. A solvent exposure technique could be employed to smooth the surfaces to mitigate this effect [40]. Alternatively, compression molding techniques provide more consistent features, despite their limitations. Also, the work presented here focuses on two-dimensional surfaces of a fixed stiffness, due to the high rigidity of PS. Stiffness is one of the main modulators of cell behavior and is well studied [60-63]. However, the PS arrays generated can be used as a mold to generate topographies and geometries on softer substrates to examine the impact of substrate stiffness.

## 5. Conclusions

Our study demonstrates the methodology and validates the first PS-based microtopography array generated by razor printing and sanding methods for cell culture studies. The cells perceived microscale surface topographies that generated measurable changes in cell morphology, growth, and phenotype. The proposed fabrication methods using tape-based razor printing [42] will enable its integration across macro and microscale culture platforms for future studies on mechanical stimulus on cell behavior.

## Supplementary Material

Figure S1 Figure S1: Effect of PS roughness in ADSC secretome.
