# Supplementary figures and images for: Polystyrene Topography Sticker Array for Cell-Based Assays
Source: Recent Prog Mater. Author manuscript; Available in PMC 2021 Mar 9. (PMC7943041; doi:10.21926/rpm.2002013)

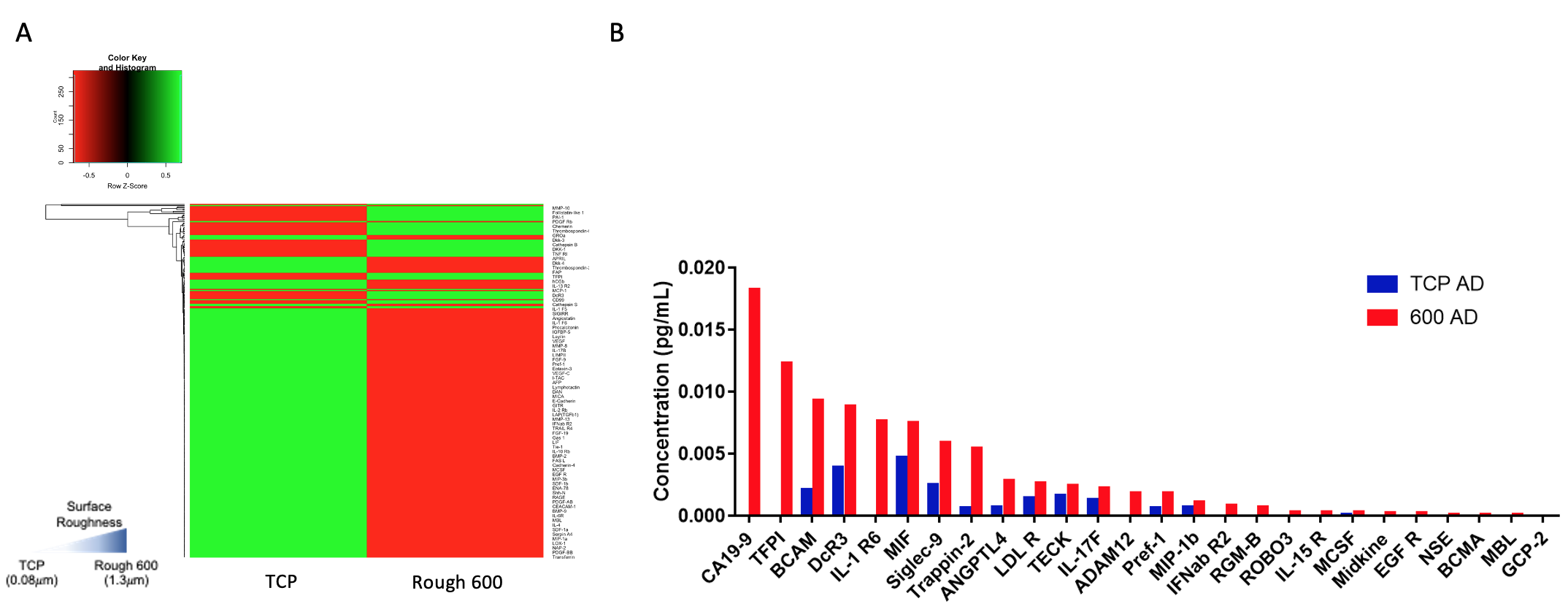

Supplement: Figure S1 — Effect of PS roughness in ADSC secretome. [file NIHMS1667541-supplement-Figure_S1.png]
